# Unusual Cause of Wheezing in a Middle-Aged Woman: A Case Report

**DOI:** 10.7759/cureus.66004

**Published:** 2024-08-02

**Authors:** Rayhan Karimi, Arun Adlakha

**Affiliations:** 1 Internal Medicine, Edward Via College of Osteopathic Medicine, Spartanburg, USA; 2 Pulmonology, Carolina Lung Clinic, Piedmont Medical Center, Rock Hill, USA

**Keywords:** recurrent pneumonia, synaptophysin, chromogranin a, lobectomy, endobronchial carcinoid tumor

## Abstract

Endobronchial carcinoid tumors, a subset of neuroendocrine tumors, represent a rare but significant entity within pulmonary neoplasms, constituting less than 2% of all lung cancers. Our case report details the clinical presentation, diagnosis, and management of a 56-year-old female patient who presented with intermittent wheezing, mucoid cough, and recurrent pneumonia. Initial imaging and bronchoscopy identified an obstructive mass in the left lower bronchus. Histopathological examination of the bronchoscopic biopsy confirmed the diagnosis of a typical endobronchial carcinoid tumor. The patient underwent a successful left lower lobe lobectomy of the lung through left thoracotomy with regional and mediastinal lymph node dissection. Follow-up evaluations demonstrated no recurrence post-treatment. This case highlights the clinical features, diagnostic challenges, and therapeutic strategies associated with endobronchial carcinoid tumors, emphasizing the efficacy of a multidisciplinary approach in achieving favorable outcomes.

## Introduction

Endobronchial carcinoid tumors are a rare subset of neuroendocrine tumors that originate in the bronchial airways. Representing less than 2% of all lung cancers, these tumors are distinguished by their relatively indolent behavior and potential for local invasion rather than distant metastasis [1]. Clinically, endobronchial carcinoid tumors can present with a variety of respiratory symptoms, including persistent cough, wheezing, recurrent pulmonary infections, and hemoptysis, often leading to misdiagnosis as more common pulmonary conditions such as pneumonia, asthma, or chronic bronchitis. The diagnosis of endobronchial carcinoid tumors is typically achieved through imaging studies, including chest radiography and enhanced computed tomography (CT) scans, followed by confirmatory bronchoscopy and biopsy. Histologically, these tumors are characterized by uniform cells with a moderate amount of cytoplasm arranged in a trabecular or rosette pattern, exhibiting neuroendocrine differentiation on immunohistochemical staining [2]. Management of endobronchial carcinoid tumors generally involves surgical resection, which can be performed bronchoscopically or through more invasive procedures determined by the tumor's size and location. Adjuvant therapies, such as radiotherapy or chemotherapy, may be considered in cases with incomplete resection or more aggressive histological features [3].

This case report aims to discuss the presentation, diagnostic approach, and therapeutic management of a 56-year-old woman diagnosed with an endobronchial carcinoid tumor, highlighting the clinical challenges and successful treatment outcomes associated with this uncommon pulmonary neoplasm.

## Case presentation

A 56-year-old Caucasian female with a past medical history of acid reflux, mild osteoarthritis, environmental allergies, hypertension, hyperlipidemia, and obesity was evaluated for a one-year history of intermittent mucoid cough, wheezing, dyspnea, and recurrent pneumonia involving the left lower lobe of the lung. She had a 15-pack-year smoking history and a family history significant for a malignant ovarian tumor in her mother. She observed blood streaking in her sputum one month prior to the evaluation, and her mucoid cough and wheezing often worsened when lying in the left lateral position. She denied chest pain, anorexia, weight loss, or night sweats. She had previously been prescribed outpatient courses of broad-spectrum antibiotics to treat three episodes of recurrent pneumonia, always involving the left lower lobe of the lung. Initially diagnosed with asthma, she was treated with inhaled steroids, nightly montelukast, and nebulized short-acting bronchodilators, but her symptoms did not improve.

**Figure 1:**
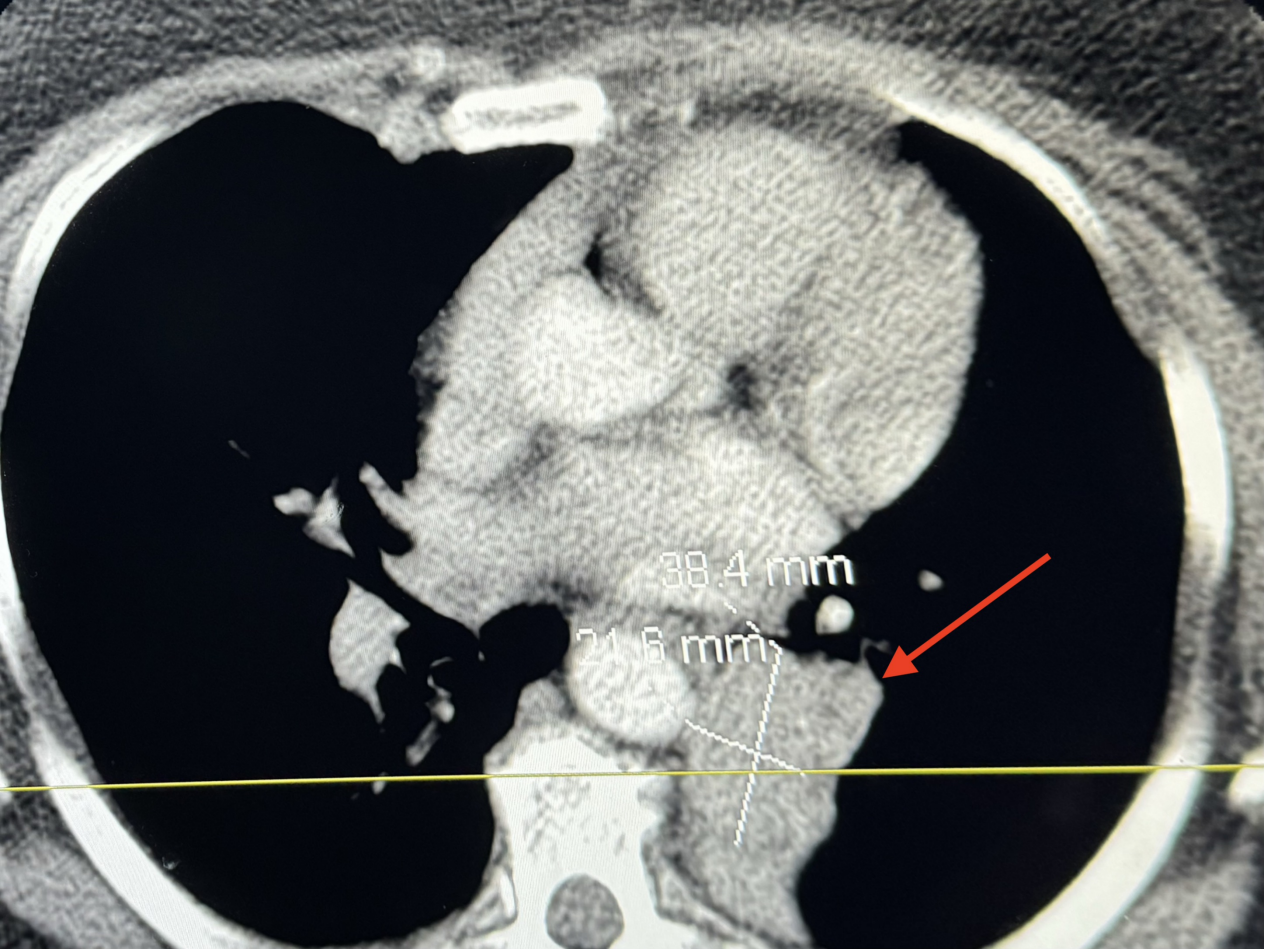
Axial CT scan showing a 38.4 mm × 21.6 mm tumor in the left lower lobe (arrow)

**Figure 2:**
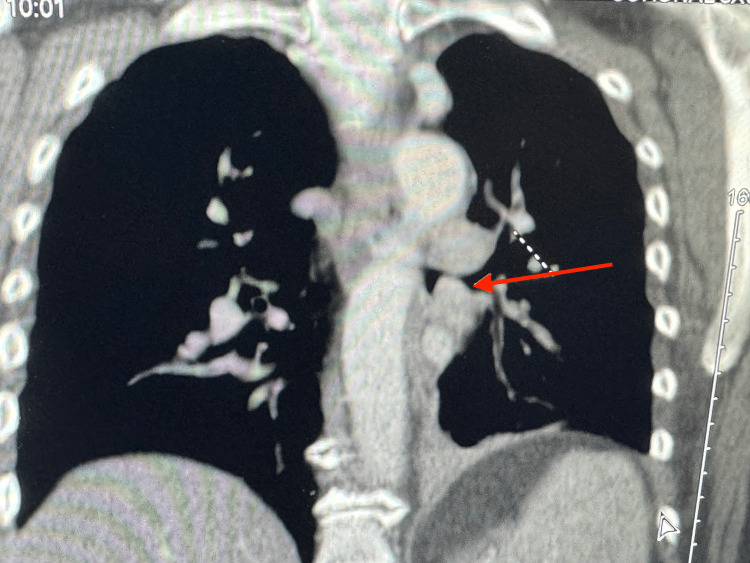
Coronal view CT scan showing a left lower lobe tumor (arrow)

**Figure 3:**
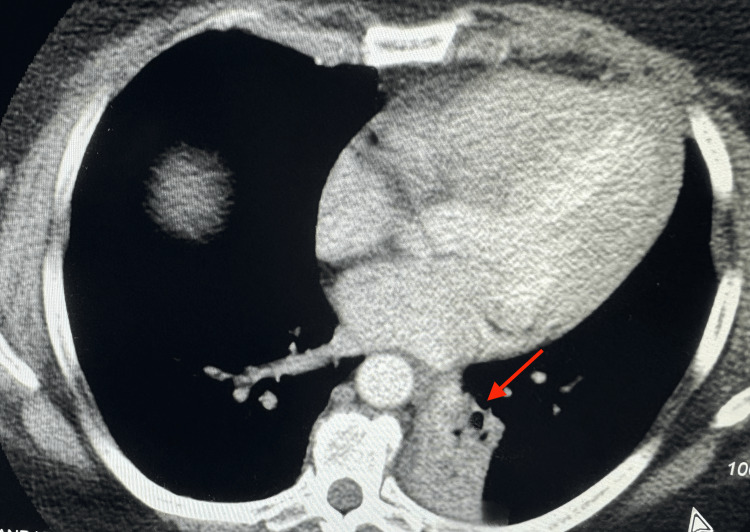
Axial CT chest displaying atelectasis of the left lower lung from the endobronchial lesion (arrow)

Physical examination revealed an obese white female with normal vital signs. The systemic exam was non-contributory except for focal wheezing and rhonchi associated with decreased breath sounds over the left infra-scapular and adjacent region, which worsened when placed in the left lateral position. Basic laboratory tests were normal. Therefore, further investigation was needed to determine the cause of the symptoms. A CT scan with contrast of the chest was completed, which revealed a soft tissue mass protruding into the left lower lobe bronchus (arrow), obliterating the bronchial lumen (Figure 1, axial view; Figure 2; coronal view). There was also associated atelectasis of the left lower lobe of the lung (Figure 3).

**Figure 4:**
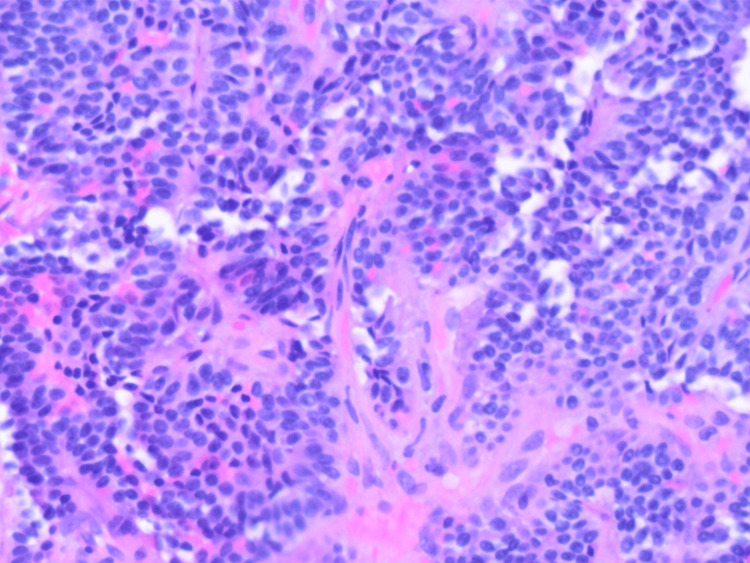
Biopsy of the tumor showing an insular growth pattern of a grade 1 neuroendocrine tumor

With positive CT chest findings, a diagnostic bronchoscopy was performed, which revealed a pinkish-red vascular mass occluding the origin of the left lower lobe bronchus, with intact overlying epithelium and a broad base attachment. Bronchial washings, brushings, and biopsies were obtained and showed proteinaceous and blood debris with reactive bronchial epithelium, but no definite neoplastic cells were found. There was no significant bleeding encountered during or after the biopsy. A left lower lobe endobronchial lung biopsy revealed an insular growth pattern of a grade 1 neuroendocrine tumor (Figure 4).

**Figure 5:**
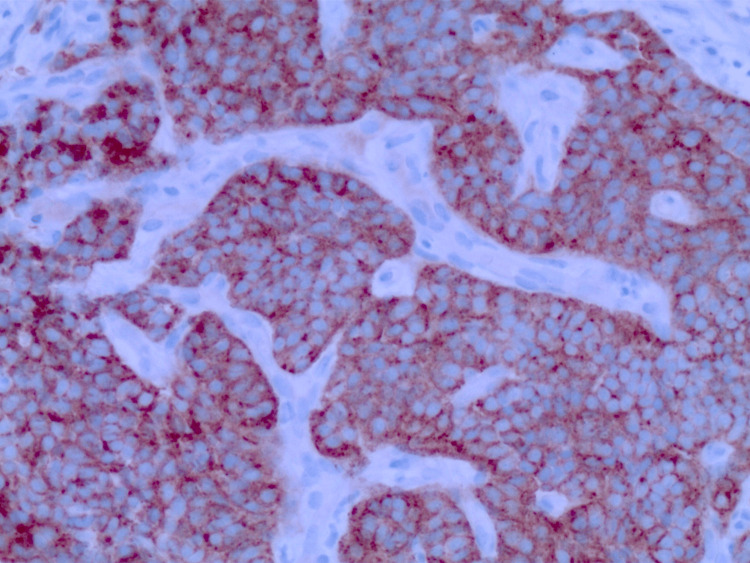
Biopsy stained for immunohistochemistry (IHC) markers positive for synaptophysin

Immunohistochemical staining showed tumor cells positive for chromogranin A (CgA) and synaptophysin (Figure 5); no malignant features, such as lymphovascular invasion, significant atypia, or mitotic activity, were noted, leading to a diagnosis of a typical bronchial carcinoid tumor. The Ki-67 index of the tumor was <3%, giving it a low grade.

**Figure 6:**
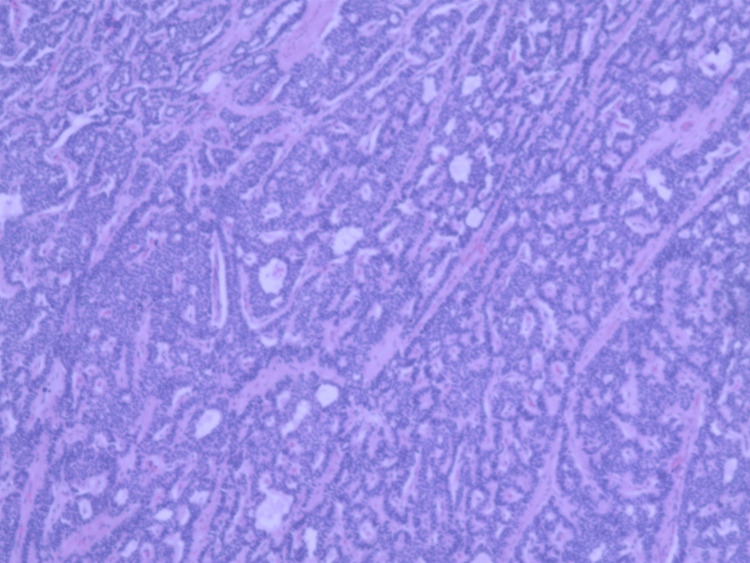
Biopsy of the resected tumor showing a well-differentiated, unifocal, typical bronchial carcinoid tumor with insular and pseudoglandular growth patterns and fine chromatin

The patient was referred to cardiothoracic surgery and medical oncology. A CT scan of the abdomen showed no evidence of metastatic disease, particularly in the liver. Serum CgA levels were low. The Tumor Board recommended a left lower lobe lobectomy with regional and mediastinal node dissection. No PET scan was performed, as there were benign histologic features along with negative CT findings. She underwent a successful left lower lobe lobectomy through left thoracotomy with no postoperative complications. Gross examination revealed an endobronchial tumor mass measuring approximately 2.9 × 2.7 × 2.5 cm with a yellow, tan homogeneous cut surface. Adjacent to the mass, seven lymph nodes ranging from 0.4 to 0.7 cm were noted, and significant mucus plugging was present. Pathology of the resected left lower lobe confirmed a well-differentiated, unifocal, typical bronchial carcinoid tumor with insular and pseudoglandular growth patterns and fine chromatin (Figure 6).

Both bronchial and vascular margins were negative for tumor involvement, and there was no evidence of visceral pleura or lymphovascular invasion. Of the nine dissected regional lymph nodes, three of the nodes adjacent to the mass were positive for tumor, with two showing direct extension, and the largest metastasis measured 7 mm. There was no extracapsular extension. No disease was noted in the bronchial or mediastinal nodes. The tumor was staged as AJCC/TNM: pT1b pN1.

Given the complete surgical resection, a tumor size of 2.9 cm in the greatest dimension, involvement of only three regional lymph nodes, and no evidence of metastasis, it was decided that she did not require adjuvant therapy. She has been closely monitored with clinical evaluations and CT scans of the chest, abdomen, and pelvis and has remained tumor-free for nearly a decade since her diagnosis.

## Discussion

Bronchial carcinoid tumors often present with nonspecific respiratory symptoms, making their early diagnosis challenging. Patients may experience persistent, intermittent cough, wheezing, and dyspnea, which are commonly misdiagnosed as more common disorders, including pneumonia, asthma, or chronic obstructive pulmonary disease. As noted in our patient, she was initially diagnosed with asthma, but her symptoms persisted despite prescribed therapy. Recurrent respiratory infections, such as pneumonia or bronchitis with or without hemoptysis, are also frequent, particularly if the tumor causes complete or partial obstruction of the airway. Additionally, some patients report chest pain or discomfort, which may be attributed to other benign conditions such as infection, inflammation, mucoid impaction, atelectasis, and adenopathy [4]. The nonspecific nature of these symptoms often results in prolonged symptom duration and delays an accurate diagnosis, emphasizing the need for maintaining a high clinical suspicion and performing a comprehensive diagnostic workup, including chest imaging and diagnostic bronchoscopy, for an explanation of persistent unexplained respiratory symptoms.

The diagnosis of bronchial carcinoid tumors involves a combination of chest imaging studies and endoscopic procedures, with bronchoscopy being the gold standard for direct visualization and biopsy of the tumor [5]. Initial imaging, typically a chest X-ray followed by a contrast-enhanced chest CT scan, is essential for identifying suspicious lesions. Compared with chest radiographs, CT provides better resolution of tumor location, tumor extent, presence or absence of mediastinal adenopathy and metastases. It gives excellent morphologic characterization of peripheral and centrally located lung neuroendocrine tumors. CT is also helpful for delineating post-obstructive atelectasis, pneumonia, mucus plugging, and mucus impaction. Other imaging procedures have a more limited role in patients suspected of having lung neuroendocrine tumors but are useful in selected cases. Baseline imaging with somatostatin receptor-based imaging techniques is generally recommended in patients with advanced neuroendocrine tumors. PET-based imaging is preferred due to its greater sensitivity. Bronchoscopy allows for direct visualization of the bronchial tree and the endobronchial lesion, enabling tissue sampling through biopsy, which is crucial for definitive diagnosis. Histopathological examination of the biopsy sample is necessary to confirm the neuroendocrine nature of the tumor, usually characterized by uniform cells with neuroendocrine differentiation on immunohistochemical staining, positive for markers such as CgA and synaptophysin [6]. Serum levels of CgA are lower with lung neuroendocrine tumors than other sites. Additionally, these levels overlap with those seen in nonmalignant conditions, giving it limited utility in localized tumors. This diagnostic approach ensures accurate identification and staging of bronchial carcinoid tumors, guiding appropriate therapeutic interventions. In our case, the CT scans were negative for metastases, and histopathology was positive for the neuroendocrine markers with the absence of malignant features, creating strong evidence for a typical endobronchial carcinoid tumor.

The WHO has created a classification system for bronchopulmonary neuroendocrine tumors, which contains two groups and four histologic categories. The first is the well-differentiated group containing the histologic categories of typical and atypical carcinoid lesions. The second is the high-grade tumors containing the histologic categories of large-cell neuroendocrine tumors and small-cell lung cancer. When one of these tumors is encountered, a Ki-67 expression index should be performed. Although it does not aid in the differential diagnosis, it creates a distinction between high-grade and intermediate-grade tumors [7].

The primary treatment for bronchial carcinoid tumors is surgical resection, which offers the best prognosis, particularly for localized tumors [8]. Surgical options range from bronchoscopic resection for smaller, well-delineated tumors to more extensive procedures like lobectomy or pneumonectomy for larger or more invasive lesions. The choice of surgical technique depends on the tumor's size, location, and extent of invasion. In cases where complete surgical resection is not feasible, adjunctive therapies such as local radiotherapy or systemic chemotherapy may be considered, though their effectiveness is less pronounced compared to surgery. Radiotherapy can be beneficial for symptom control or in cases of residual disease post-surgery. Somatostatin analogs, such as octreotide, may be used in managing symptomatic patients, particularly those with hormonally active tumors, to control symptoms and potentially slow tumor growth [9]. Advanced cases with metastatic disease might require a combination of surgical, medical, and targeted therapies to manage the disease comprehensively. Overall, a multidisciplinary approach involving thoracic surgeons, medical and radiation oncologists, pulmonologists, and pathologists is crucial for optimizing treatment outcomes for patients with bronchial carcinoid tumors.

Carcinoid syndrome (CS) is a clinical condition that arises from the release of vasoactive substances, such as serotonin and histamine, by carcinoid tumors, most commonly when these tumors metastasize to the liver [10]. This syndrome is characterized by a distinct set of acute symptoms, including episodic flushing, diarrhea, abdominal pain, bronchospasm, and, in persistent, severe cases, development of long-term sequelae, including venous telangiectasias, retroperitoneal fibrosis, and carcinoid heart disease, which causes valvular lesions primarily on the right side of the heart. The episodic flushing typically affects the face and upper chest, and the diarrhea can be debilitating, significantly impacting the patient's quality of life [11]. With the presence of a carcinoid tumor, the incidence of developing CS increases, specifically in GI carcinoid tumors. In our case, the serotonin produced by the tumor in the lung is degraded by the liver. Therefore, there were no signs of CS. CS is often a late manifestation of the disease, indicating that the tumor has metastasized and has bypassed hepatic metabolism to enter the systemic circulation. Lung neuroendocrine tumors produce less quantity of serotonin than midgut tumors and account for lower rates of CS. CS is most often encountered with tumors >5 cm or extensive liver metastasis. Diagnosis is typically confirmed by elevated levels of urinary 5-hydroxyindoleacetic acid (5-HIAA), a metabolite of serotonin [12]. Management of CS involves controlling symptoms with somatostatin analogs like octreotide or lanreotide, which inhibit the release of serotonin and other hormones. In more advanced cases, interventions, such as hepatic artery embolization, peptide receptor radionuclide therapy, and surgical resection of liver metastases, may be considered to reduce tumor burden and alleviate symptoms.

In a review of our described case, patients with recurrent or ongoing pulmonary symptoms despite optimal medical therapy, it is recommended they undergo a CT scan of the chest to evaluate for underlying etiology. Additionally, a follow-up diagnostic bronchoscopy should be performed to assess for any endobronchial pathology.

## Conclusions

This case report highlights the diagnostic and therapeutic challenges associated with bronchial carcinoid tumors, emphasizing the importance of considering this uncommon entity in patients with persistent, nonspecific respiratory symptoms and recurrent pneumonia. Symptoms are usually from the obstructing tumor mass or bleeding due to hypervascularity. Patients with these tumors usually have received several courses of antibiotics to treat recurrent pneumonia before a diagnosis is made. Respiratory symptoms and signs accentuated by certain positions of the body, as well as pneumonia repeatedly involving the same segment or lobe of the lung (both noted in our patient), should alert the physician to the presence of endobronchial pathology. The case of our 56-year-old woman underscores the necessity of thorough diagnostic evaluations, including advanced imaging and bronchoscopy, to achieve an accurate diagnosis. Surgical resection remains the cornerstone of treatment, offering the best prognosis for localized disease. The successful management of this patient's tumor through a multidisciplinary approach led to a favorable outcome, with no recurrence nearly a decade post-surgery. This reinforces the effectiveness of current treatment protocols and the importance of long-term follow-up. Awareness and early recognition of bronchial carcinoid tumors can significantly improve patient outcomes, highlighting the need for continued education and research in this field.
